# Observation of neutron emission during acoustic cavitation of deuterated titanium powder

**DOI:** 10.1038/s41598-024-62055-6

**Published:** 2024-05-21

**Authors:** Max Fomitchev-Zamilov

**Affiliations:** Maximus Energy Corporation, Naples, FL USA

**Keywords:** Nuclear energy, Physics

## Abstract

Possibility of nuclear reactions in solid state is intriguing for two reasons: (1) It provides a means of studying nuclear processes in conditions that are much different from traditional plasma-filled reactors or particle accelerators; (2) it dramatically lowers the cost and complexity of the experimental setups by eliminating the highly capital intensive components such as plasma/vacuum systems and particle accelerators. In this article we report the observation of neutron emission coincident with acoustic cavitation of deuterated titanium powder suspended in mineral oil. The resulting neutron emission was detected using an assembly of ^3^He proportional neutron counters. The peak neutron count rate was in excess of 6500 CPM, more than 10,000 times in excess of background. The observed neutron emission was coincident with the application of acoustic influence. The neutrons were present only when secondary acoustic waves originating from the complex bubble interactions inside the reactor constructively interfered resulting in massive, sharp pressure peaks on the order of a few thousand psi. We were able to sustain the neutron production for several hours and repeated the experiment multiple times under various conditions. We hypothesize that the observed neutrons originate from nuclear fusion of deuterium ions dissolved in titanium lattice due to the mechanical action of the impinging cavitation jets, although other processes (such as spallation) still need to be ruled out.

## Introduction

Nuclear fusion is universally deemed to be the future of power generation. Unfortunately, numerous technological challenges and high costs associated with conventional fusion approaches—inertial and magnetic confinement—still place the commercial fusion power far in the future. This impasse provides ample motivation for exploration of alternative fusion approaches. Unfortunately, deep skepticism surrounding much publicized (and consequently much scandalized) ‘cold fusion’ and ‘bubble fusion’ deterred most serious academics from pursuing alternatives. Fortunately, private enterprises appear to be less constrained but they seldom publish their advances for commercial reasons. We seek to break with this ‘tradition’ and hereby report our findings with the goal to excite the academic community with the possibility of practical nuclear reactions in condensed matter.

## Automated nuclear lab

**Figure 1:**
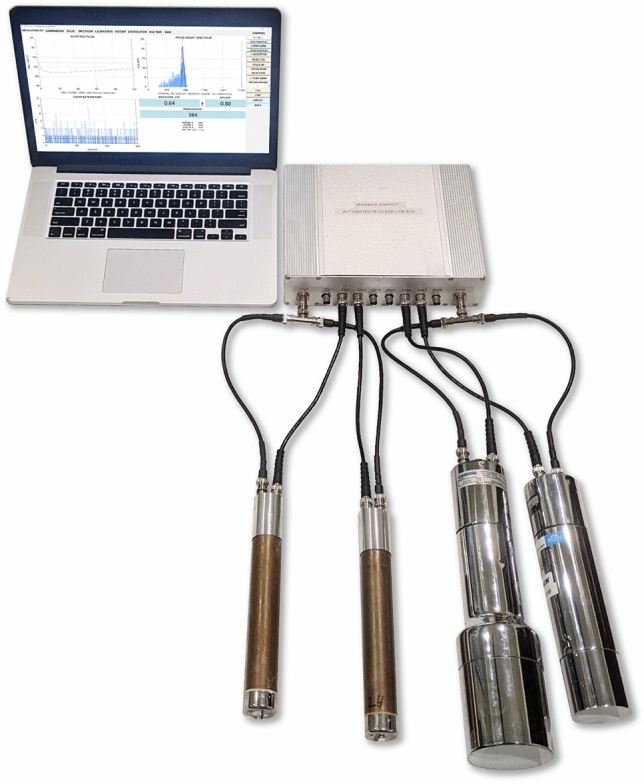
The Automated Nuclear Lab system: sample configuration with two ^3^He proportional neutron counters and two NaI(Tl) gamma scintillation detectors.

The initial motivation for this work came from our interest in observing neutron emission resulting from nuclear fusion that was hypothesized to occur within the cores of rapidly collapsing deuterium bubbles under the influence of an intense acoustic drive^1^. Therefore, our first order of business was the development of a versatile and highly reliable neutron detection system. After several years of intense R&D we have completed and made commercially available the Automated Nuclear Lab (ANL) hardware and PulseCounter Pro software—Fig. 1—the tools essential for rapid nuclear experimentation^2^.

The key design features of the ANL are:

- Simultaneous acquisition of nuclear data from multiple instruments.
- Raw detector signal logging and storage.
- Flexible pulse processing algorithm that allows for effective noise discrimination.
- Real-time display, comprehensive inspection, and statistical analysis of results.
- Possibility of the offline experiment reanalysis under a different set of assumptions.

## Neutron detection

**Figure 2:**
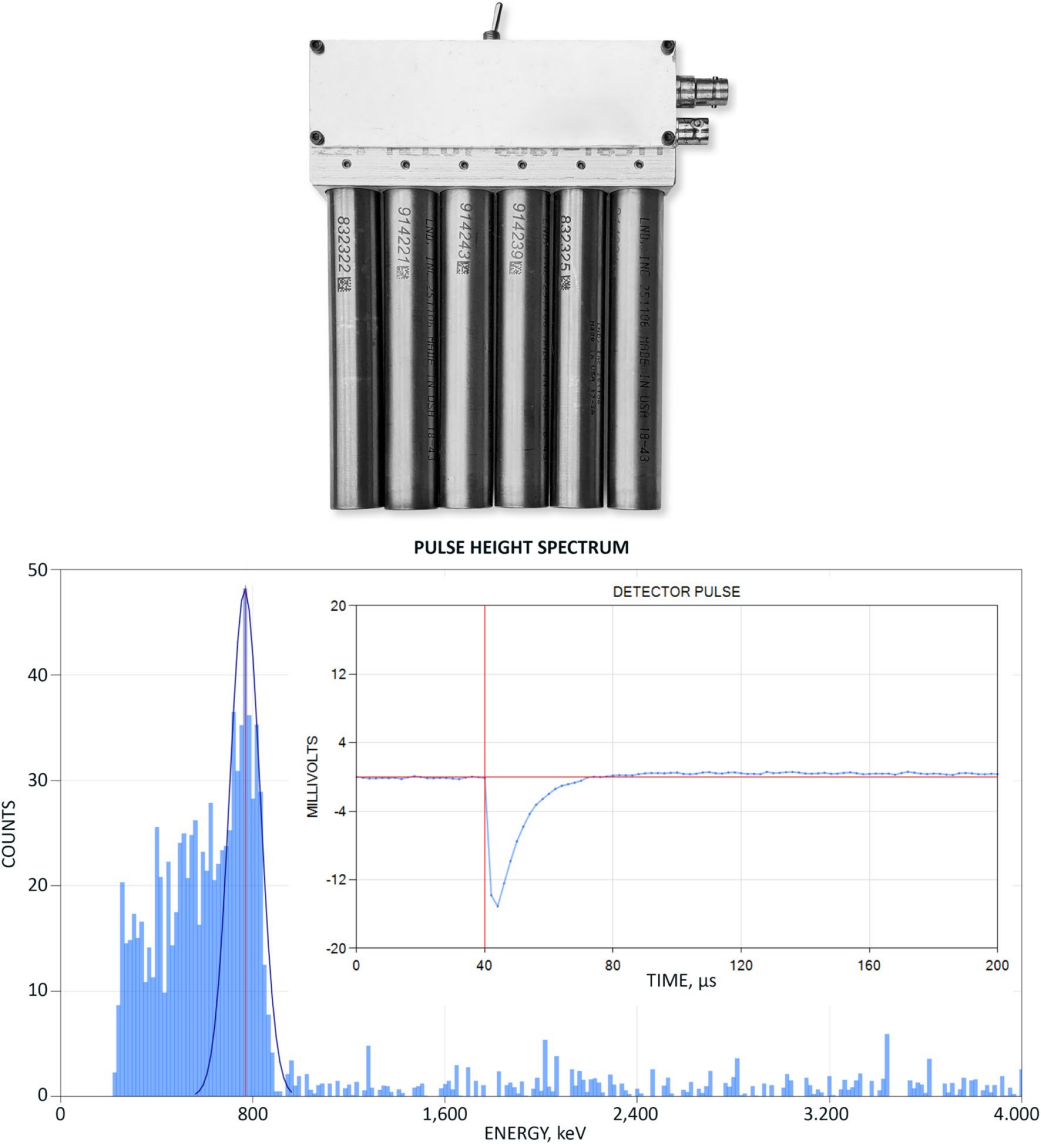
(Top) The bank of six LND 251106 ^3^He proportional counters; (Bottom) its thermal neutron spectrum; (Inset) a typical detector pulse.

As a primary tool for neutron detection we chose ^3^He-filled proportional counters due to their high sensitivity and the easily recognizable shape of the thermal neutron spectrum that they produce. In most experiments we used an assembly of six LND 251106 detectors arranged in a bank as shown on Fig. 2

The detector bank did not contain a preamp, therefore we used a 1250 V detector bias to maximize the output signal, which was AC-coupled via a 100 pF capacitor to an ANL input channel. A typical pulse height was ~ 10 mV and a typical pulse width was ~ 30 μs, Fig. 2(Inset). The thermal neutron spectrum obtained using a Po-Be ‘check’ source is shown on Fig. 2b. The detector signal was captured at 500 kHz with 16-bit resolution. The pulse height spectrum was obtained by measuring the pulse height with respect to the zero-volt baseline. The pulse rejection threshold was set to 1.5 mV to discount pulses due to gammas or noise.

A typical neutron counting protocol used in out experiments was as follows:

- Acquire a ‘calibration’ measurement using a 5 mCi Po-Be ‘check’ source; the objective of the ‘calibration’ measurement is to tune the pulse processing algorithm to reject gammas and electromagnetic noise until a satisfactory thermal neutron spectrum is obtained.
- With the experimental setup complete and the neutron detector in its intended place acquire the ‘background’ counts over an extended period of time (typically 10–60 min, but sometimes as long as 24 h); the ‘background’ counts acquired over a sufficiently long period of time must form a thermal neutron spectrum and the count rate histogram must fit a Poisson distribution.
- With the experimental setup operating acquire the ‘experiment’ counts; the ‘experiment’ counts must also form a thermal neutron spectrum, however the count rate Histogram may not fit a Poisson distribution as the neutron emission during the experiment is not necessarily random; if the resulting neutron spectrum deviates from thermal, steps must be taken to eliminate noise (e.g. eliminate ground loops, reduce capacitive coupling, eliminate mechanical contact and vibration, etc.) until the spectrum shape corresponds to a thermal neutron spectrum.
- For very low count rate experiments we employ a borax castle to reduce background counts to below 1 CPM; in this case there may be not enough counts to form a continuous spectrum, therefore we manually examine each counted pulse to make sure that the pulse shape, width and rise time match the expected detector response.
- During the course of an experiment we may record several ‘background’ and several ‘experiment’ measurements, each such measurement comprising a sequence of count rate samples acquired over a desired period of time; to derive conclusions PulseCounter Pro software conducts statistical analysis by applying Student’s T-test to the population of the ‘experiment’ and the ‘background’ count rates and calculates a P-value; P-values less or equal to 0.05 are programmed to report the difference between the population means as statistically significant.

We use the ANL and PulseCounter Pro software to execute the above mentioned protocol. This greatly improves productivity and thus enables rapid nuclear experimentation.

## Experimental setup

**Figure 3:**
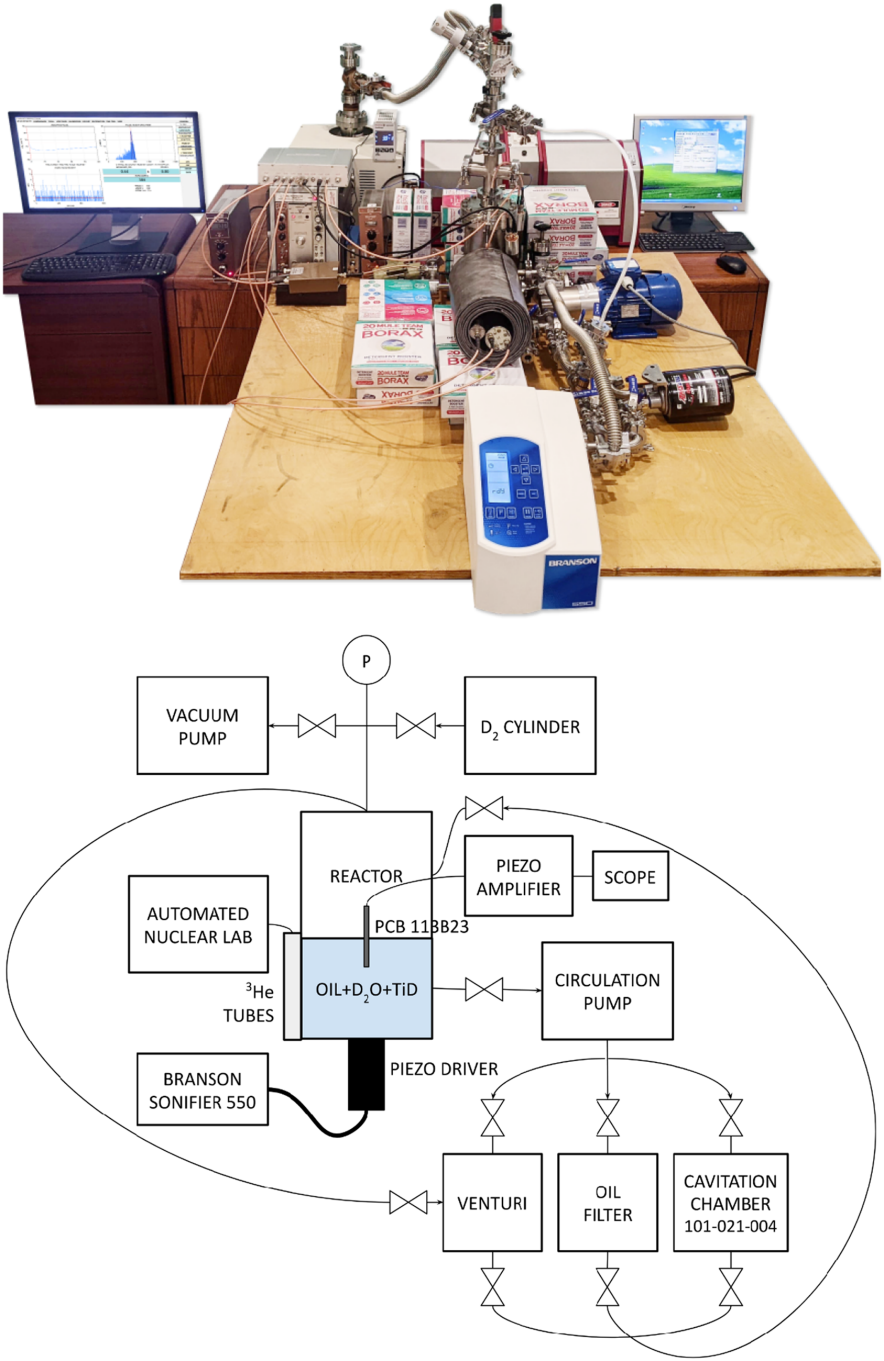
(Top) The experimental setup; (Bottom) its block diagram.

Our experimental setup and its block diagram are shown on Fig. 3.

The reactor was made out of a 6″ stainless conflat vacuum tee section with a Branson Sonifier piezoelectric driver (20 kHz/550 Watt max) attached to the bottom blank plate. The reactor was connected to a recirculation loop comprising a venturi nozzle, a 2 micron oil filter (Amsoil EABP90) and a secondary cavitation chamber, which was used for fluid preparation/conditioning before the experiment.

The neutron detector bank was situated in the back of the reactor without making physical contact with the reactor wall. We used acoustic foam to protect the detector assembly from acoustic noise and in later experiments we employed a grounded copper plate to reduce electromagnetic interference due to capacitive coupling between the reactor and the detector assembly.

The reactor headspace was connected to a turbomolecular vacuum pumping station used for degassing. A Pfeiffer PCR280 compact Pirani capacitance pressure gauge was used to monitor the headspace pressure.

The sound within the reactor was monitored by a PCB Piezotronics 113B23 piezoelectric transducer connected to a digital oscilloscope and/or to a second data acquisition channel of the ANL system.

Particle size distribution was measured by the Sympatec HELOS Magic laser diffraction system, which was connected to the reactor via a separate side chain fitted with a compact magnetic gear pump to push the reactor fluid through a Sympatec Sucell inline measurement cell coupled to the HELOS laser.

The reactor fluid was mineral oil equivalent to MultiTherm IG-4. For most experiments the oil was thoroughly degassed via cavitation under vacuum until the reactor headspace pressure dropped below 1E−4 Torr.

For the experiments characterized by a significant neutron flux the reactor fluid was a suspension of a deuterated titanium powder (Ti/D) combined with various quantities of a heavy water (D_2_O) microemulsion. This working fluid was prepared by cavitation of the oil with a few hundred milligrams of the Ti/D powder and approximately 1 mL of D_2_O in the Branson 101-147-048 cup horn for several minutes until homogeneity was achieved. Then various quantities of this suspension/emulsion were introduced into the reactor and thoroughly mixed with the reactor oil using the recirculation loop with the filter disengaged.

The Ti/D powder was prepared as follows: a stainless steel cup cathode with 100 g of commercial-grade 500 mesh 99.8% Ti powder was electrochemically loaded with deuterium using a Ni plate at 2.5 V for 80 h in a dilute (0.05 M) boric acid in D_2_O electrolyte.

## Results

In the initial set of experiments we focused on detecting neutron emission associated with rapidly collapsing deuterium bubbles in mineral oil under the influence of acoustic drive. To create the bubbles we have filled the reactor headspace with pure deuterium gas while circulating the oil through the venturi nozzle. We have conducted hundreds of tests runs trying various ambient bubble sizes (from 100 nm to 100 micron), various ambient pressure (from 1E−4 to 1000 Torr), various acoustic drive amplitude (from 1 to 10,000 psi), various acoustic frequencies (from 20 to 100 kHz) and various surfactants (SDS, Triton-X, PFA) but failed to detect any neutron emission above background level. We used a borax castle to reduce background counts to below 1 CPM and on some experiments employed up to twelve ^3^He neutron counters 1.25″ diameter by 8″ encircling the reactor for nearly 2π solid angle coverage (the reactor top and bottom plate as well as the viewport were not covered). Yet we failed to observe even a 1% increase in neutron counts above background during these experiments.

We have also tried various Xe + D_2_ mixtures as suggested in Ref.^3^ but with the same lack of excess neutrons.

For the second set of experiments we shifted away using bubbles and switched to D_2_O droplets. As before, we have systematically combed the parametric space while varying the droplet concentration from extremely concentrated (the oil was opaque and milky in appearance) to extremely dilute (the oil was clear in appearance, but we could detect the droplets using the HELOS laser). Once again we did not detect any excess neutrons at 1% level above background.

**Figure 4:**
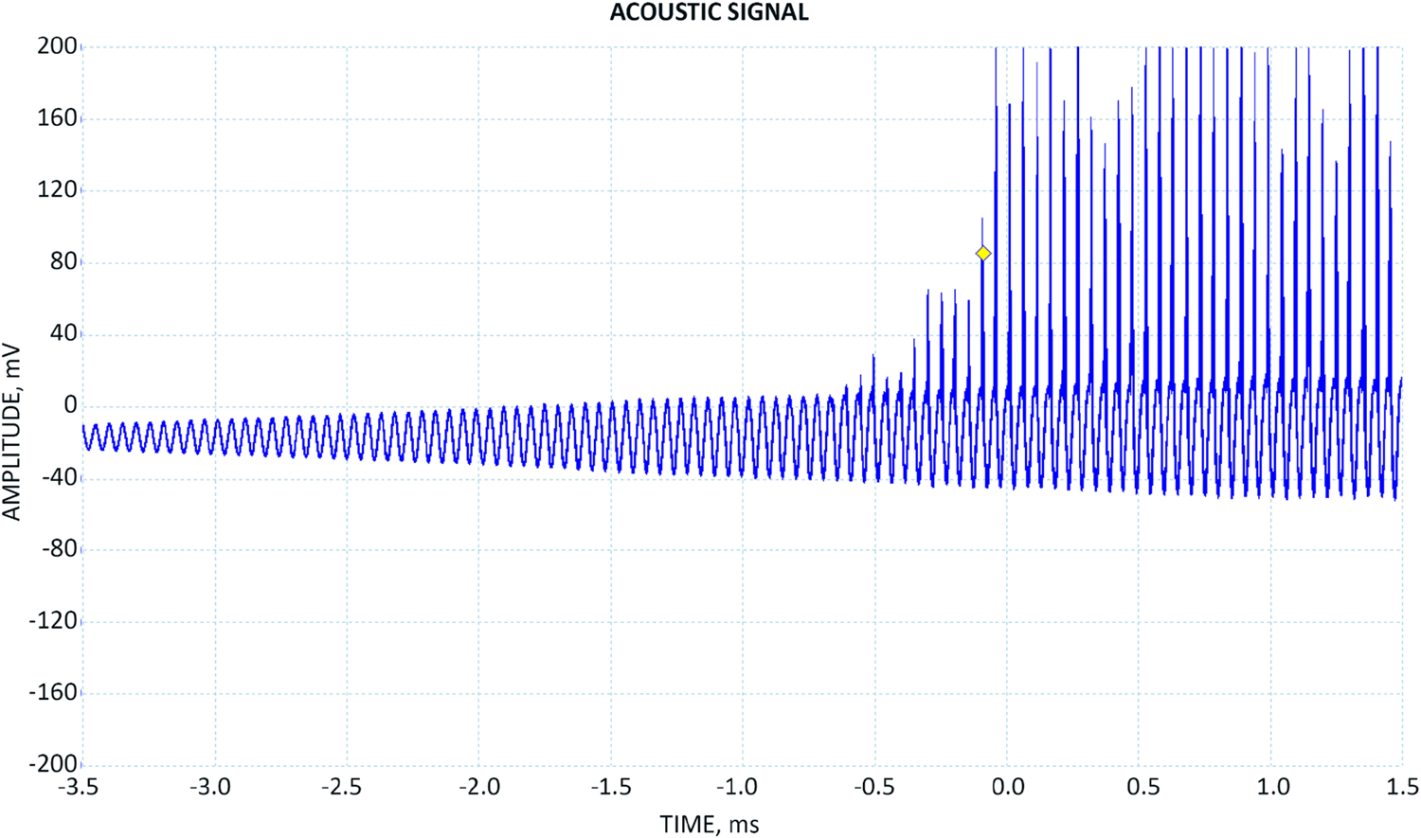
An acoustic signal from the reactor: small-amplitude 20 kHz acoustic wave corresponds to the external acoustic drive applied to the reactor, clipping acoustic pulses are thought to arise from constructive interference of the outgoing shockwaves launched by the rebounding bubbles.

While experimenting with the droplets we made the following observations:

1. Microdroplets in oil are exceedingly stable against cavitation and degassing; even intense (> 1000 psi amplitude) acoustic drive under vacuum was very inefficient in converting droplets into bubbles.
2. Cavitation was all but absent even in presence of surfactants; we did not detect significant levels of cavitation noise under *most* conditions.
3. We could remove the microdroplets only by filtering.
4. Sometimes, when the droplet size was matched with the amplitude and frequency of the acoustic drive we observed stupendous ‘secondary’ acoustic peaks that we hypothesize originated from constructive interference of the outgoing shockwaves originating from rebounding or oscillating bubbles^4^ (Fig. 4).

These extreme peaks sometimes were in excess of 24,000 psi to where they saturated the PCB transducer and the actual pulse magnitude could not be read. Several times the transducer got damaged. Two times the 6″ quartz viewport of the reactor got cracked or completely shattered. It is hard to imagine what sort of acoustic magnitude was achieved inside the reactor to make this destruction possible. The peaks tended to be stronger and easier to obtain when the emulsion concentration was very high and the reactor oil was milky in appearance.

During the third set of experiments we introduced samples of suspension that contained both Ti/D particles and D_2_O droplets into the reactor and after thorough mixing subjected the working fluid to a 20 kHz acoustic drive at maximum power with the 0.01 s on/0.01 s off duty cycle. Almost immediately we registered a significant neutron flux that occasionally exceeded 200 CPS/12,000 CPM (we did not use a borax castle and the background counts were below 0.5 CPS/30 CPM). This happened when a large suspension sample (~ 0.5 L) was introduced into the reactor at ambient pressure and the reactor oil consistency was milky in appearance. The actual neutron flux could have been even higher since we did not use any moderator around our detectors on the account that the reactor oil itself will thermalize neutrons originating from within the reactor.

**Figure 5:**
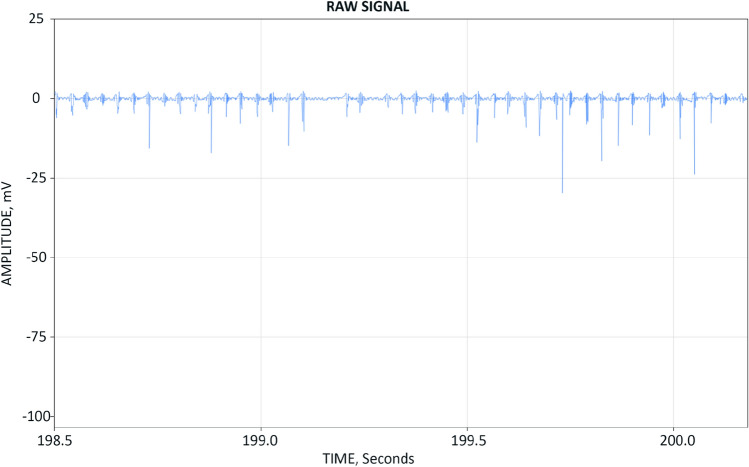
The raw neutron detector signal; neutron events (thin vertical lines) correlate neatly with EM noise (low magnitude high frequency oscillations below the neutron detection threshold level) due to capacitive coupling between the neutron detector bank and the piezoelectric driver; the noise period coincides with the 0.01 s on/off piezoelectric driver duty cycle.

When we examined the raw detector signal (Fig. 5) we observed numerous neutron events evenly spaced in time. The neutron event clusters correlated neatly with the acoustic drive duty cycle. specifically, the neutron events predominantly occurred when the acoustic drive was on. This was evident through the EM noise that leaked into the detector signal due to capacitive coupling between the reactor and the detector bank. This noise appeared as low magnitude high frequency oscillations below the neutron detection threshold level.

We were able to repeat this high-flux experiment several times in the course of two days with similar results.

To further study the phenomenon we changed the experimental protocol as follows:

1. We started introducing just 1 mL of suspension into the reactor in order to obtain consistently repeatable results under better controlled conditions for a systematic study.
2. We switched to using the neutron detector bank depicted on Fig. 2 as with it we could use a grounded copper sheet to break the capacitive coupling between the reactor and the detector bank and thus eliminate the EM interference from the piezoelectric driver and thus capture a much cleaner detector signal: the copious amounts of neutrons, EM noise and numerous overlapping multi-neutron events from the previous experiments prevented us from obtaining a clean thermal neutron spectrum, which we felt was necessary to observe in order to remove all doubts about the nature of the emission.
3. We used borax castle to reduce the background counts to below 1 CPM.

**Figure 6:**
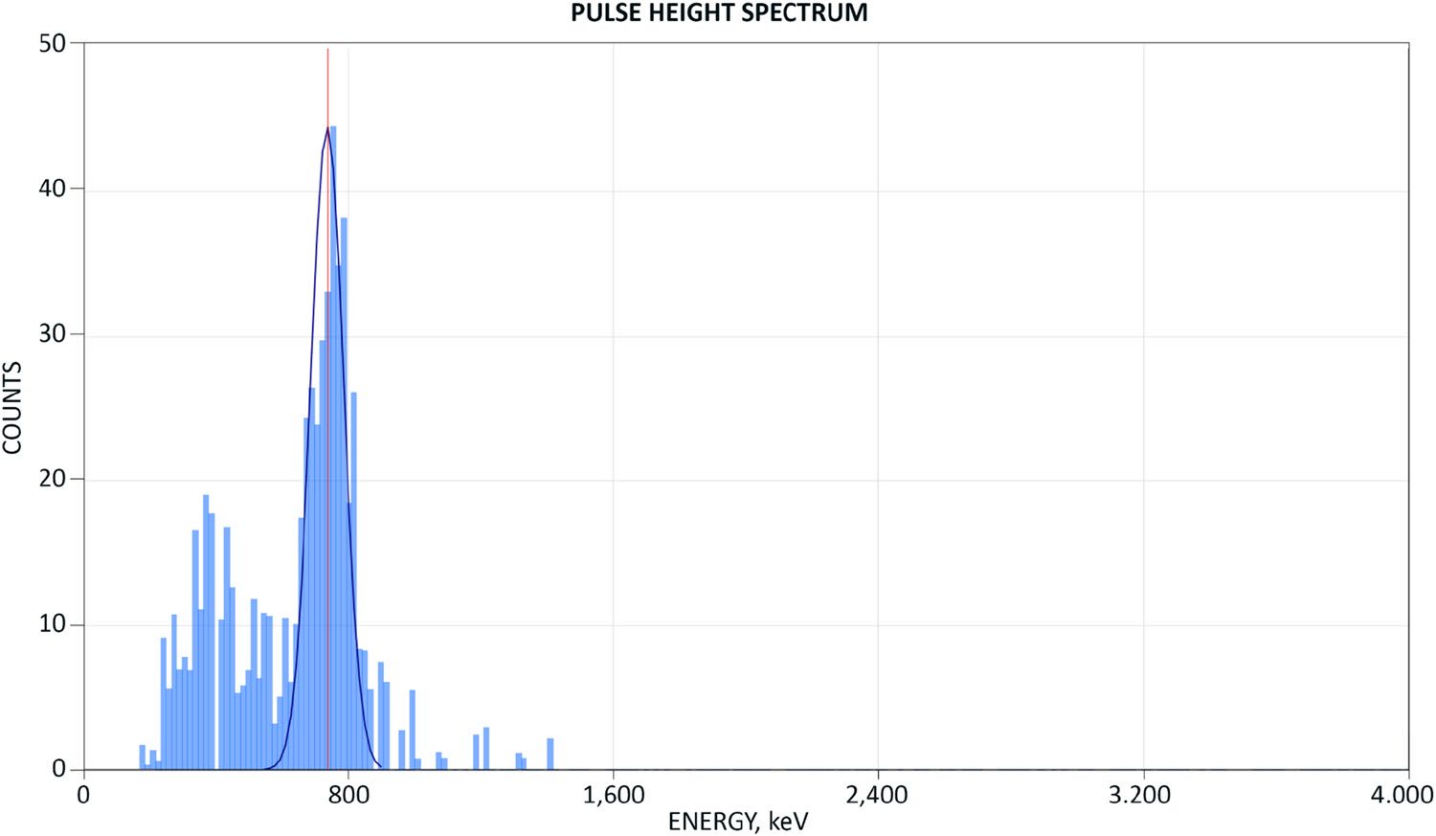
The clean thermal neutron spectrum arising from the neutron flux from the reactor.

As a result of the change of protocol we have been able to conduct numerous successful experiments in the following months and captured a clean thermal neutron spectrum depicted on Fig. 6.

The spectrum on Fig. 6 was acquired during 23 min of the reactor operation. We have captured 1497 count rate samples, recorded and stored the raw detector signal in its entirety. The mean count rate during the experiment was 0.2 CPS/12 CPM; the mean background count rate (which was established by sampling the background for 20 h) was below 0.01 CPS/0.6 CPM. The difference between the ‘experiment’ and ‘background’ mean count rates in this particular example is 20× and is highly statistically significant (p < 0.000).

During another similar 34 min run the mean ‘experiment’ count rate was 1 CPS/59.4 CPM or 100 times in excess of the background.

**Figure 7:**
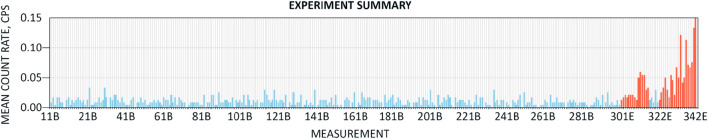
The mean count rates of a long sequence of 4 min measurements: the blue bars correspond to the ‘background’ (reactor off) measurements, the red bars represent the ‘experiment’ (reactor on) measurements; note that the counts briefly return to the background level when the reactor is turned off during the measurements 317 to 321.

Figure 7 depicts the plot of the mean count rates of 300 four-minute ‘background’ measurements (20 h total), followed by 16 four-minute ‘experiment’ measurements, followed by further 5 ‘background’ measurements ending with the additional 21 ‘experiment’ measurements. The tenfold jump in the count rates when the acoustic drive is activated is clearly evident.

**Figure 8:**
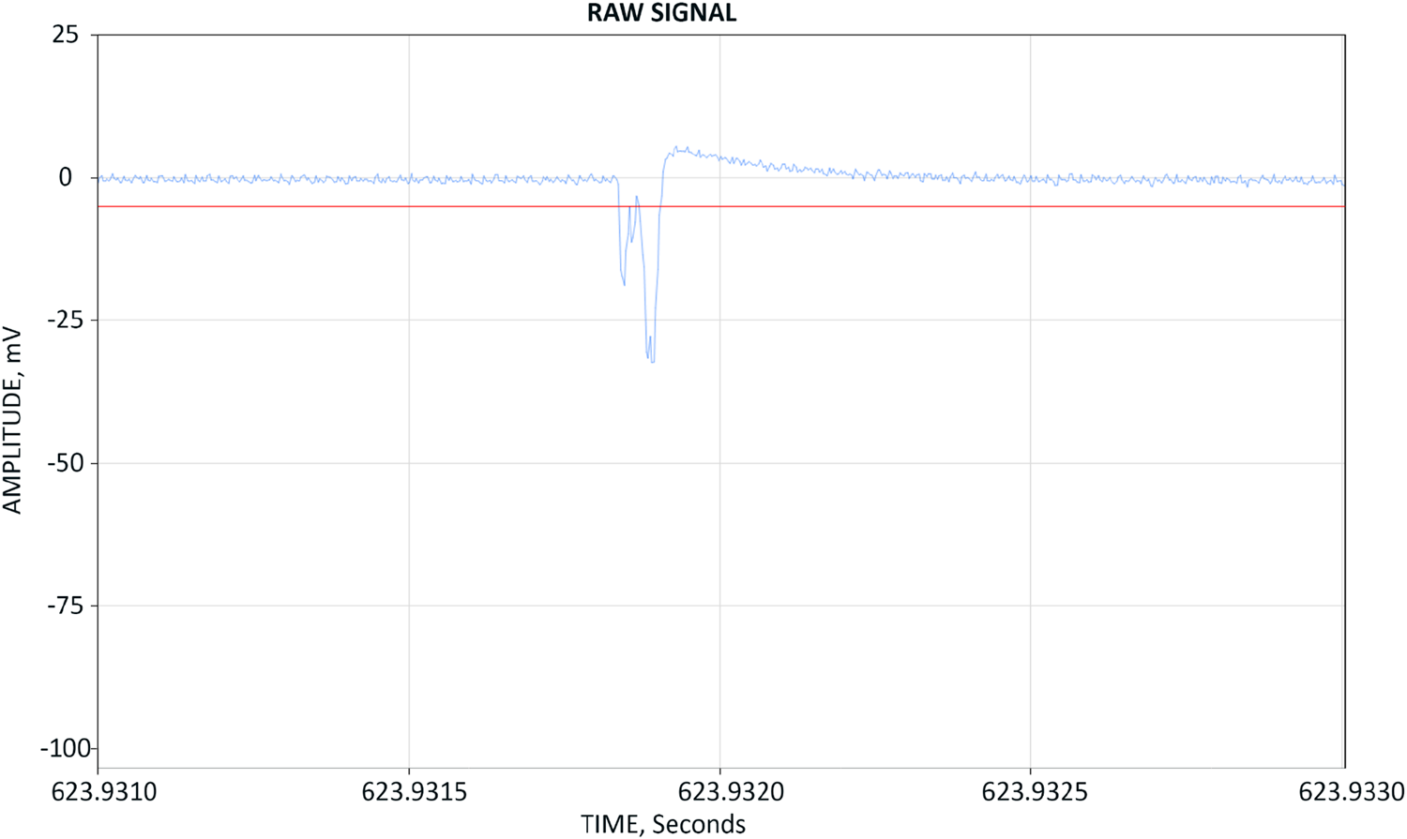
Multiple overlapping neutron events.

In some experiments we observed multiple neutron events that are spaced so tightly that the software algorithm could not resolve their pulse height accurately (Fig. 8). When this happened the resulting neutron spectrum got predictably distorted.

Our attempts to measure gamma emission from the reactor using a Bicron 2M2 2″ NaI(Tl) scintillator connected to the ANL yielded counts and spectra consistent with the background. The detector was placed in immediate proximity with the reactor wall but without making physical contact. Due to the relatively low neutron flux it is possible that the accompanied gamma flux was too weak to be detected due to absorption in the reactor oil and in the reactor wall.

## Conclusions and outlook

At this point we have barely scratched the surface and cannot claim the full understanding of the observed phenomenon. Yet the results appear interesting and novel enough to report. While we are preparing for the new round of study we are happy to share all the raw data that we acquired during the discussed experiments with any interested researchers. The data can be opened using the PulseCounter Pro software or can be imported into 3rd party software tools.

On the basis dozens of successful experiments our conclusions are as follows:

1. We did not detect any excess neutrons at 1% level above the background from cavitation of D_2_ bubbles or D_2_O droplets in mineral oil under any conditions, however extreme; we conclude that thermonuclear fusion within the collapsing bubbles is unlikely because it is virtually impossible to ensure a perfect spherical symmetry of the bubble collapse, and the slightest deviation from the symmetry results in the development of an instability in the form of a cavitation jet that pierces the bubble during the final stages of the collapse^5^.
2. We observed a significant neutron flux (more than 6500 CPM or 10,000 times in excess of the background) coincident with the acoustic influence when we introduced a suspension of deuterated titanium powder into the reactor.
3. We were able to repeat the experiment many times over the course of 6 months and sustained the neutron production for 1.5 h during the longest run.
4. The neutron flux was present only when massive (few thousand psi) acoustic peaks were registering inside the reactor.
5. We hypothesize that the massive acoustic peaks arise from the constructive interference of the outgoing shockwaves resulting from the rebounding or oscillating bubbles.
6. The neutron flux was not present when the Ti/D powder was absent from the reactor.
7. The created suspension of the Ti/D powder in oil was stable against decay and separation for at least 6 months.
8. We hypothesize that the observed neutron flux is caused by cavitation jets impinging on Ti/D solids causing DD fusion within the bulk or on the surface of the particles; however, we do not yet know the exact role of the jets as there are two distinct possibilities that come to mind: (a) the jets act as a ‘pistons’ compacting deuterium ions stored in the titanium lattice, in which case the material of the jets is not that important and H_2_O droplets should be as effective as D_2_O droplets; (b) the jets must contain deuterium ions and the mechanism of action is that of an ion beam impinging on a deuterated target; in this case D_2_O is crucial and H_2_O droplets are not be expected to produce the reaction.
9. At this stage we cannot rule out other sources of neutrons such as spallation; additional spectroscopic studies are necessary in order to establish the hypothesized thermonuclear nature of the observed neutrons.

## Data Availability

The datasets used and/or analyzed during the current study available from the corresponding author on reasonable request. The datasets include raw counts and raw detector signals for all the discussed measurements accessible via PulseCounter Pro software, which can also be exported into other formats.
