# Author Correction: Observation of neutron emission during acoustic cavitation of deuterated titanium powder

**DOI:** 10.1038/s41598-024-67868-z

**Published:** 2024-07-19

**Authors:** Max Fomitchev‑Zamilov

**Affiliations:** Maximus Energy Corporation, Naples, FL USA

Correction to: *Scientific Reports* 10.1038/s41598-024-62055-6, published online 21 May 2024

The Acknowledgments section in the original version of this Article was incomplete.

“The authors thank Alan Smith for preparation of the deuterated titanium powder. We acknowledge the financial support of Maximus Energy Corporation in conducting this research.”

now reads:

“The authors thank Alan Smith for preparation of the deuterated titanium powder. We acknowledge the financial support of Maximus Energy Corporation in conducting this research and thank Ross Koningstein for financial assistance with publishing this paper.”

The original Article has been corrected.

